# Texture analysis on muscle T2 maps can reveal changes in upper trapezius muscles related to primary headache disorders

**DOI:** 10.1038/s41598-026-63292-7

**Published:** 2026-07-27

**Authors:** Nico Sollmann, Paul Schandelmaier, Corinna Börner-Schröder, Severin Schramm, Jonathan Stelter, Thomas Baum, Gabby B. Joseph, Kornelia Kreiser, Meinrad Beer, Claus Zimmer, Dimitrios C. Karampinos, Florian Heinen, Michaela V. Bonfert, Michael Dieckmeyer

**Affiliations:** 1 Department of Diagnostic and Interventional Radiology, University Hospital Ulm, Ulm, Germany; 2 Department of Diagnostic and Interventional Neuroradiology, School of Medicine and Health, TUM Klinikum Rechts der Isar, Technical University of Munich, Munich, Germany; 3 TUM-Neuroimaging Center, TUM Klinikum Rechts der Isar, Technical University of Munich, Munich, Germany; 4 Department of Nuclear Medicine, University Hospital Ulm, Ulm, Germany; 5 Department of Pediatric Neurology and Developmental Medicine, LMU Hospital, Dr. von Hauner Children’s Hospital, Munich, Germany; 6 LMU Center for Children with Medical Complexity, Ludwig Maximilian University, iSPZ Hauner, Munich, Germany; 7 Department of Diagnostic and Interventional Radiology, School of Medicine and Health, TUM Klinikum Rechts der Isar, Technical University of Munich, Munich, Germany; 8 Department of Radiology and Biomedical Imaging, University of California San Francisco, San Francisco, CA USA; 9 Laboratory of Magnetic Resonance Imaging Systems and Methods, Ecole Polytechnique Federale de Lausanne (EPFL), Lausanne, Switzerland; 10 Center for Biomedical Imaging (CIBM), Lausanne, Switzerland; 11 Department of Diagnostic, Interventional, and Pediatric Radiology, Inselspital, University of Bern, Bern, Switzerland

**Keywords:** Migraine, Tension-type headache, Trapezius muscle, T2 mapping, Texture analysis, Trigemino-cervical complex, Diseases, Health care, Medical research, Neurology, Neuroscience

## Abstract

Migraine and tension-type headache (TTH) are highly prevalent primary headache disorders. Neck pain is common among patients with migraine or TTH, but objective biomarkers for the neck’s myofascial involvement within the trigemino-cervical complex (TCC) are lacking. This study aimed (1) to apply texture analysis (TA) to magnetic resonance imaging (MRI) covering the upper trapezius muscles with T2 maps, which are particularly sensitive to subtle edematous changes such as those following inflammation; and (2) to associate demographics, characteristics of sleep and sports, headache, and neck pain with muscle T2 values and derived texture features. Seventy-one participants (mean age 24.0 ± 3.0 years, 59 females; 22 healthy controls [HC], 21 migraine, 16 TTH, 12 migraine with TTH) underwent MRI of the upper trapezius muscles, followed by bilateral muscle segmentations and extraction of muscle T2 and first- and second-order texture features. Comparisons between HC and participants with primary headache disorders were performed following removal of outliers with the robust regression followed by outlier identification (ROUT) method (Mann-Whitney U tests, adjustment for multiple comparisons). Principal component analysis (PCA), linear, and logistic regression analyses (using bootstrapping with 5,000 permutations) were conducted based on image-derived parameters and headache and neck pain characteristics. Significant group differences were found between HC and participants with primary headache disorders for muscle T2 (30.14 ± 1.05 vs. 31.10 ± 0.92 ms, *p* < 0.001), sumaverage (0.007163 ± 0.0011 vs. 0.006366 ± 0.0012, *p* = 0.017), kurtosisglobal (1.15 ± 0.32 vs. 1.63 ± 0.81, *p* = 0.024), and energy (0.003514 ± 0.0006 vs. 0.004176 ± 0.0012, *p* = 0.043). According to PCA, a cluster including age, days with headache over the last three months, neck pain, muscle T2, kurtosisglobal, and skewnessglobal was identified, with a cluster close to the opposite direction including average hours of sports per week, average hours of sleep per week, sex, and varianceglobal. Muscle T2 (*p* = 0.015) and sumaverage (*p* = 0.022) were significantly associated with days with headache over the last three months. Furthermore, sumaverage was significantly associated with the presence of neck pain (*p* = 0.024). T2 mapping with TA may provide objective and quantitative parameters for myofascial involvement and peripheral sensitization within the TCC, partially associated with clinical parameters such as the number of days with headache.

**Electronic supplementary material:**

The online version of this article (10.1038/s41598-026-63292-7) contains supplementary material, which is available to authorized users.

## Introduction

According to the Global Burden of Disease Study 2021, primary headache disorders such as migraine or tension-type headache (TTH) are ranked 15th for causes of global disability-adjusted life-years, with a count of 48.0 million^1^. Regarding years lived with disability (YLDs), primary headaches are ranked 3rd, thus making up 5.2% of all-cause YLDs^1^. On a high rank for YLDs is neck pain with 2.2% of all-cause YLDs, which has shown coincidences with primary headache disorders^1–4^. For instance, in a study among secondary school students using questionnaire assessments, associations of neck pain with headache were primarily observed for migraine and migraine plus concomitant TTH^5^. Furthermore, disability due to neck pain was observed in 69% among episodic migraine and 92% among chronic migraine, with time since migraine onset having a high influence^6^. However, despite evidence of strong ties between headache and neck pain in primary headache disorders, their relationship remains incompletely understood, and objective biomarkers for this relationship are lacking, which could potentially facilitate misdiagnosis or suboptimal treatment and patient management^7–9^.

Pathophysiological concepts converge central and peripheral mechanisms of pain perception, processing, perpetuation, and sensitization in migraine and TTH^7–9^. For the peripheral component, neck musculature plays a central role, activated by stimuli such as muscle contraction, strain, or inflammation^7–12^. The nociceptive sensation in the periphery from myofascial tissue is then conveyed via thin myelinated (Aδ) and unmyelinated (C) afferent fibers, running within the C1 to C3 spinal nerves to the trigeminocervical nucleus^7–9,13^. Information is further processed with trigeminal afferent inputs, transmitted onto second-order neurons, and further transferred to the trigemino-thalamic tract and linked to the brain’s pain processing regions^7,8,10–12^. This pathway is referred to as the trigemino-cervical complex (TCC), which may work as a bidirectional conceptualization incorporating myofascial structures^13–15^. In this regard, the nociceptive inflow could further trigger neurogenic inflammation by retrograde excretion of key substances such as calcitonin gene-related peptide, which may maintain and exaggerate peripheral sensitization^7,8,10–12^. In the context of the TCC, TTH and migraine may lie within a semiological continuum, with several cranial nerves and the greater occipital nerves as potential sources or input for head pain^16–19^.

Given the lack of an objective biomarker for the myofascial involvement in primary headache disorders, the reference standard remains manual palpation of muscles, including identification of myofascial trigger points (mTrPs)^20^. However, this approach may lack sufficient reproducibility and reliability, and it cannot provide scalable or quantitative biomarkers. In this regard, quantitative magnetic resonance imaging (MRI) could help investigating skeletal musculature, with the aim of providing objective biomarkers for myofascial involvement. Particularly T2 mapping could allow to identify and quantify changes in myofascial tissue as related to subtle neurogenic inflammation. It has been applied to imaging of the upper trapezius muscles^21–23^, given that the trapezius muscles are afferently innervated by anterior rami of spinal nerves (C3-C4 via cervical plexus) and thus may be closely connected to the concept of the TCC^7–9,13^. It has been shown by T2 mapping that T2 values of the trapezius muscles were significantly higher in subjects with migraine as compared to healthy controls (HC), as well as in subjects with TTH or TTH plus migraine when compared to HC^21,22^. Furthermore, mTrPs of the trapezius muscles as defined by manual palpation showed potential correlates on T2 maps with significantly increased focal T2 values among patients with migraine^23^. Further, elevated muscle T2 may be primarily related to released endogenous inflammatory mediators that could cause subtle edema in myofascial tissues^7,24–26^.

Yet, T2 mapping does not necessarily capture the entirety of changes in myofascial tissue related to primary headache disorders, given that it provides a T2 value for a certain region of interest (ROI) as the primary measure. Texture analysis (TA) is an advanced image analysis method that enables extracting more quantitative information contained in medical imaging data^27–31^. As a subfield of radiomics, TA uses mathematical methods to quantify the spatial variation of voxel intensities in medical imaging data, thus providing objective assessment of tissue characteristics^27–31^. When applied to T2 mapping from MRI, it could allow identifying changes in myofascial tissue beyond mere muscle T2 values or visual perception, and thus yield additional potential biomarkers of the myofascial involvement in primary headache disorders.

Against this background, this study applied TA to T2 maps from MRI covering the upper trapezius muscles in patients with primary headache disorders, with the aim of identifying objectifiable changes in myofascial tissues as compared to HC. In addition, we aimed to assess potential associations of characteristics of headache, neck pain, demographics, and sleep and sports with muscle T2 values and derived texture features.

## Materials and methods

### Study design, inclusion, and exclusion criteria

This prospective monocentric study was approved by the local ethics committee and conducted in accordance with the Declaration of Helsinki (registration numbers: 154–12 & 5679/13 & 193/19S, Ethikkommission der Technischen Universität München, Munich, Germany). Written informed consent was obtained prior to study participation.

This study’s protocol included manual palpation of the neck muscles, MRI acquisitions of the neck region focused on the upper trapezius muscles, assessment of neck pain, and assessment of headache, sports, and sleep characteristics. Between the manual palpation and MRI acquisition, a two-week interval was established^21,22^. All study visits took place between July 2019 and August 2020. Participants of this study have been previously investigated for different purposes, i.e. to demonstrate increased muscle T2 values of the trapezius muscles in subjects with migraine, TTH, or migraine plus TTH compared to HC (without TA)^21,22^.

The inclusion criteria for this study were as follows: (1) age between 18 and 30 years; (2) a diagnosis of migraine only (with or without aura), TTH only (TTH- group), mixed-type headache (TTH and migraine, TTH+ group), or absence of any history of headache disorders in participants considered as HC. The classification of headache disorders was done according to the German version of the headache questionnaire modified according to the diagnostic criteria of the third edition of the International Classification of Headache Disorders (ICHD)^32^. Specifically, a participant was diagnosed with migraine if recurrent attacks (lasting 4 to 72 h) were present, with typical characteristics of unilateral location, pulsating quality, moderate or severe intensity, aggravation by routine physical activity, and association with nausea and/or photophobia and phonophobia^32^. In migraine with aura, recurrent attacks with also unilateral fully-reversible visual, sensory, or other central nervous system symptoms had to be present^32^. Furthermore, a participant was diagnosed with TTH if typically bilateral, pressing or tightening pain of mild to moderate intensity was present^32^.

The exclusion criteria were as follows: (1) any history of muscular or neurological disorders (except for the respective headache diagnosis); (2) a diagnosis of any other primary headache (e.g., cluster headache); (3) any history of previous injury, surgery, or implants at the neck region; (4) participation in competitive sports, extensive physical activity, or weightlifting/body building; (5) intake of muscle relaxants; (6) any interventions for neck pain such as massage or physiotherapy in the two weeks prior to the MRI exam; (7) a body mass index (BMI) indicating underweight or obesity (BMI < 18.5 or BMI > 30.0 kg/m^2^); (8) contraindications for MRI; (9) pregnancy.

### Assessment of cohort and headache characteristics, neck pain, and myofascial trigger points

Participant characteristics including demographics (such as age, sex, and BMI [in kg/m^2^]) were documented, as well as average days with acute analgetic drug intake per month and the individual primary substance taken. Specifically, the self-reported weekly frequency of sports activity, habitual sleep duration on weekdays, and the number of days per month with analgesic drug intake were recorded by customized questionnaires. No standardized instruments were used for these self-reported measures.

Furthermore, participants with primary headaches as well as HC reported the number of days with headache over 90 days preceding the study appointment. Additionally, on the day of assessment, the presence of neck pain was self-reported and categorized as present or absent. Participants were classified as having no neck pain if they had not experienced neck pain within the preceding 30 days. Pain intensity and functional impairment were not assessed.

Within the upper trapezius muscles, the overall number and locations of mTrPs were identified by manual palpation by a certified physiotherapist^21,33,34^. This examination did not take place on the day of MRI acquisitions (to avoid potential bias of muscle T2 values in relation to manual palpations). We considered latent (criteria: palpable taut band with a local hypersensitive spot; local hypersensitive spot with occurrence of a referred sensation during palpation; palpable taut band with a local hypersensitive spot and occurrence of a referred sensation during palpation) and active mTrPs (criterion: referred sensation of a hypersensitive spot reproducing typical headache symptoms)^33–36^.

### Muscular magnetic resonance imaging

#### Image acquisition

One 3-Tesla MRI scanner with a 16-channel anterior coil, a 12-channel built-in-the-table posterior coil, and a 16-channel head coil was used for scanning of the neck and shoulder region (Ingenia Elition, Philips Healthcare, Best, The Netherlands). Participants were scanned in supine position and – regarding participants with a primary headache diagnosis – in their inter-ictal phases (i.e., on days without any headaches). The scan protocol included a T2-prepared three-dimensional (3D) turbo spin-echo (TSE) sequence for T2 mapping and a T2-weighted DIXON TSE sequence for anatomical co-registration. The pulse sequence protocol for T2 mapping is shown in Supplementary Table 1.

#### Image segmentation

The T2-prepared 3D TSE sequence for T2 mapping was processed with in-house developed MATLAB scripts (version R2021a; MathWorks Inc., Natick, MA, USA), using a voxel-by-voxel fitting approach while additionally accounting for B0 field inhomogeneities^21–23,37^. Visual quality control and segmentation were performed with MITK (version 2022.04; www.mitk.org). No images had to be excluded due to compromised image quality (e.g., due to severe motion artifacts).

**Fig. 1:**
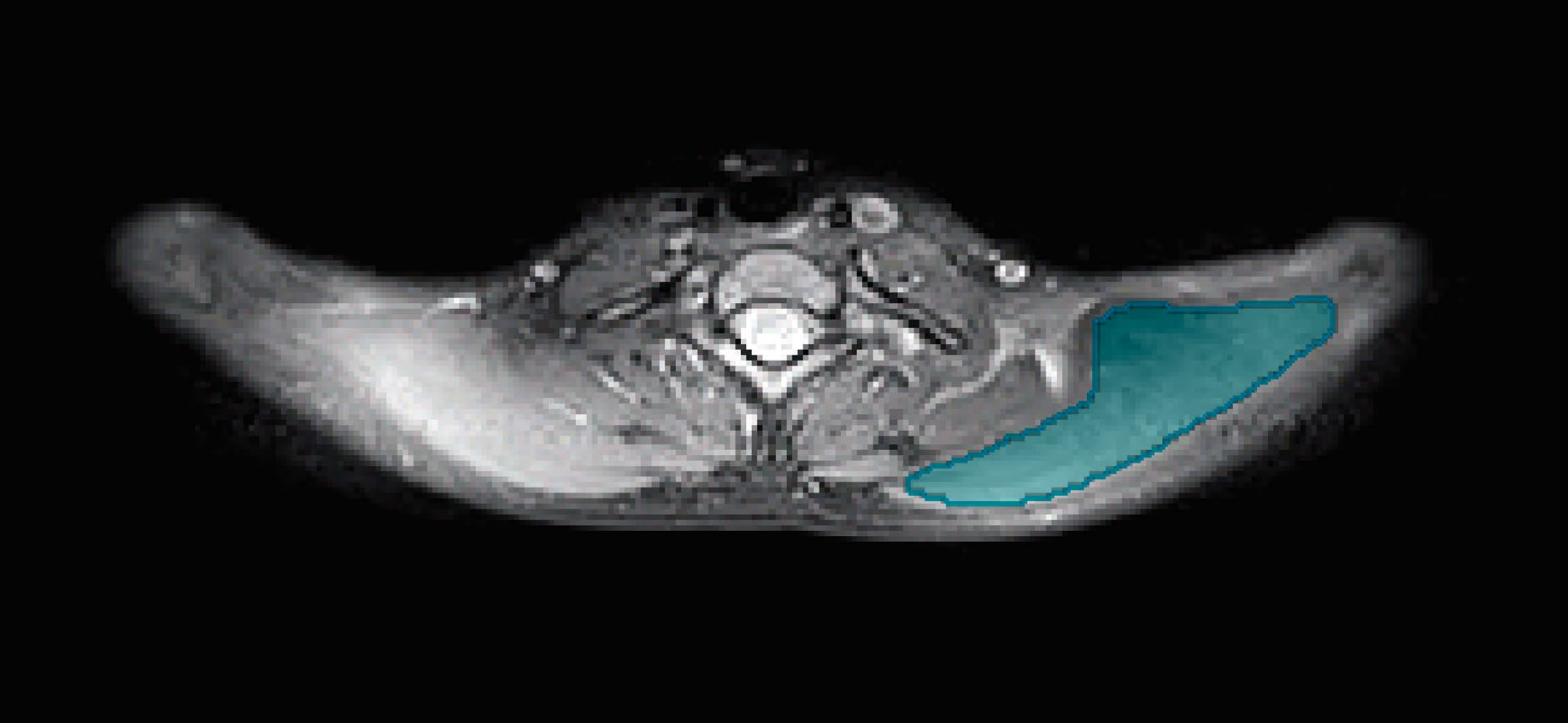
Exemplary unilateral segmentation of the upper trapezius muscle on one representative axial magnetic resonance imaging (MRI) slice, with the segmented part being shown in turquoise color. For the purpose of this study, bilateral muscle segmentations were performed as the basis for extraction of muscle T2 values and derived texture features.

On the images with the shortest T2 preparation duration, the entire upper trapezius muscles (as far as captured by imaging and discriminable) were segmented bilaterally on axial slices by placing and contouring polygonal ROIs (Fig. 1)^21,22^. A margin of approximately 5 mm was considered at the outer muscle contours to avoid accidental inclusion of muscle fascia or intermuscular fat within the ROIs. The ROI placements were completed by one reader, blinded to group assignments as well as presence and locations of mTrPs. High reproducibility of those manual segmentations with T2 extractions (root-mean-square coefficient of variation [RMSCV]: 0.12 ± 0.07%), as well as high inter-reader reliability (RMSCV: 1.43 ± 0.64%) have been shown previously^22^.

#### T2 extraction and texture analysis

Using the in-house developed scripts for MATLAB, the mean T2 values of the left and right trapezius muscles were extracted from the ROIs, and thresholded for T2 values above 100 ms (i.e., voxels with T2 values > 100 ms were excluded, given that high T2 values may most likely stem from areas of very high fluid content components, such as vasculature)^21,22,38^.

Additionally, TA was run on the ROIs of the T2 maps by using MATLAB with a radiomics toolbox (https://github.com/mvallieres/radiomics). Based on distributions of gray-level values, different texture features were quantified to characterize structural image properties^27–31^. Specifically, different first-order statistical moments from global gray-level histograms as well as second-order features based on the gray-level co-occurrence matrix (GLCM) were extracted^39–42^. The GLCM indicates how often pairs of voxels with a given gray-level value and offset are present within an image or ROI. The GLCM entries at different angular directions (i.e., θ = 0°, 45°, 90°, and 135°) were computed by the joint probability of two adjacent voxel intensities at a given offset (d = dx, dy, dz) and given θ. In this context, dx, dy, and dz denote the displacements along the x-, y-, and z-axis, respectively. Furthermore, the GLCM analysis used computation of the co-occurrence probabilities of voxel intensities from the 26 neighbors aligned in 13 directions. The direction-dependent discretization length differences were considered when measurements were combined by averaging or summation, and averaging over the 13 directions ensured rotation invariance. Table 1 provides an overview of the extracted texture features and their common interpretations.

**Table 1:** Texture features from texture analysis (TA) based on T2 maps from magnetic resonance imaging (MRI) of the upper trapezius muscles, including their respective common interpretations^39,43,44^.

Texture feature	Category	Interpretation
Varianceglobal	First-order texture features / global texture features	Gray-level distribution – spread
Skewnessglobal		Gray-level distribution – shape
Kurtosisglobal		Gray-level distribution – flatness
Energy	Second-order texture features	Uniformity
Contrast		Local intensity variation
Entropy		Randomness
Homogeneity		Homogeneous scene
Correlation		Linear spatial relationships between texture elements
Sumaverage		Spread of the mean voxel co-occurrence distribution
Variance		Voxel co-occurrence distribution
Dissimilarity		Heterogeneity

### Statistical analysis

Statistical analyses were performed using Prism (version 10.6.1; GraphPad Prism, Boston, MA, USA), SPSS (version 31.0.0.0; IBM Corp., Armonk, NY, USA), and Excel (version 2508; Microsoft Corp., Redmond, WA, USA). A p-value < 0.05 was considered statistically significant.

The muscle T2 values as well as first-order texture features (i.e., varianceglobal, skewnessglobal, kurtosisglobal) and second-order texture features (i.e., energy, contrast, entropy, homogeneity, correlation, sumaverage, variance, dissimilarity) derived from the ROIs covering parts of the upper trapezius muscles were averaged to obtain a mean value for both sides together. Descriptive statistics including mean and standard deviation (SD), absolute or relative frequencies, and/or 95% confidence intervals (CIs) were calculated for participant characteristics, including information on headache and neck pain as well as hours spent with sleep or sports, as well as for muscle T2 values and first- and second-order texture features. For the T2 values and texture features, outliers were detected and systematically removed using the robust regression followed by outlier identification (ROUT) method (with a Q-value of 5%)^45^. Outlier removal was achieved separately per each of the twelve image-based parameters. All following analyses used the datasets with outliers removed.

For demographics and cohort characteristics, group comparisons were performed with Mann-Whitney U tests or Fisher’s exact tests. Given that at least some of the first- and second-order texture features were not normally distributed according to Kolmogorov-Smirnov tests, for group comparisons Mann-Whitney U tests were chosen regarding muscle T2 values and first- and second-order texture features. Group comparisons on those MRI-derived measures were performed for HC vs. participants with migraine, TTH-, and TTH+ together, for HC vs. participants with migraine plus TTH+ together, as well as within participants with primary headache disorders separately for first- and second-order texture features (i.e., migraine vs. TTH-, migraine vs. TTH+, TTH- vs. TTH+, and migraine plus TTH + vs. TTH-). For those group comparison tests, adjustments for multiple comparisons were performed separately using the Benjamini-Hochberg procedure with an accepted false discovery rate (FDR) of 15%^46^. Additionally, for muscle T2 values and the first- and second-order texture features that showed statistically significant differences in analyses for HC vs. participants with migraine, TTH-, and TTH+ together, receiver operating characteristics (ROC) with the area under the curve (AUC) were calculated (based on the Wilson/Brown method).

A principal component analysis (PCA) was computed, which is a multivariate approach to reduce a dataset’s dimension while retaining as much information as possible (i.e., to summarize multivariate data into a limited number of dimensions)^47,48^. This was done for the entire cohort using the following parameters: age, sex, BMI, neck pain, days with headache in the last three months, number of mTrPs, average hours of sports per week, average hours of sleep per week, muscle T2, first-order texture features, and second-order texture features (considering the datasets after outlier removal). A PCA model on standardized data was chosen, in which variables were first transformed so that each variable has a mean of 0 and SD of 1 (i.e., all variables are set on the same scale, which implies that in the identified principal components [PCs] each variable is weighted equally). The PCs were selected based on parallel analysis, which performs Monte Carlo simulations (on “random” data of equal dimensions to the input data) and calculates eigenvalues for all resulting PCs, using a percentile level of 90% and 5,000 simulations. In this parallel analysis, the number of PCs was chosen by determining the point at which the PCs were indistinguishable from those generated by simulated data. Related to the obtained PCs, loadings (i.e., the correlation or covariance between columns of data and PCs, allowing identification of clusters of variables), proportions of variance, eigenvalues (from the data and from parallel analysis), and eigenvectors were extracted.

Furthermore, for those texture features with a statistically significant group difference, linear regression was performed in the whole cohort with the respective texture feature and the days with headache over the last three months or the number of mTrPs (with pairwise case exclusions regarding missing values due to outlier removal). Moreover, logistic regression was performed with the respective texture feature and the presence of neck pain (as a binary parameter). Those regression analyses were adjusted for age, sex, and BMI using a two-block design (hierarchical linear or logistic regression), and bootstrapping with 5,000 permutations was applied.

## Results

### Participant characteristics

Overall, 71 participants were included in this study: 22 HC, 21 participants with migraine, 16 participants with TTH (TTH-), and 12 patients with migraine and a concomitant diagnosis of TTH (TTH+). In the study cohort, 45 participants with primary headache disorders were right- and 4 left-handed, and none of them reported any other neurological disorders. Regarding acute analgesics, participants with primary headache disorders took on average on 3.22 ± 2.95 days / month their major analgetic drug, with non-steroidal anti-inflammatory drugs being most commonly used among acute medications. Time since first occurrence of the primary headache disorder was on average 9.06 ± 5.38 years (*n* = 32). In females among participants with a primary headache disorder, the menstrual cycle duration was on average 20.76 ± 16.51 days (*n* = 37).

Table 2 provides an overview of additional cohort characteristics, also including details on headache and neck pain. Detailed characteristics for the patient group with primary headache disorders can be found in Supplementary Table 2.

**Table 2:** Cohort characteristics. This table shows demographics as well as headache and neck pain characteristics for the participants with migraine, tension-type headache only (TTH-), and tension-type headache plus migraine (TTH+) as well as healthy controls (HC). Data are shown as mean ± standard deviation (SD) or absolute / relative frequencies.

	HC (*n* = 22)	Migraine / TTH- / TTH+ (*n* = 49)	*p*-value
Age (mean ± SD, in years)	23.0 ± 2.2	24.4 ± 3.3	0.180
Sex (females)	17 (77.3%)	42 (85.7%)	0.495
BMI (mean ± SD, in kg/m^2^)	22.0 ± 2.3	21.8 ± 2.0	0.939
Neck pain (present)	0 (0%)	30 (61.2%)	< 0.001
Days with headache (in the last three months, mean ± SD)	3.0 ± 2.3 (*n* = 21)	20.3 ± 16.8	< 0.001
Number of mTrPs (all, mean ± SD)	4.0 ± 4.0	4.0 ± 2.9	0.532
Hours of sports (per week, mean ± SD)	4.1 ± 2.1	2.8 ± 1.5	0.009
Hours of sleep (per week, mean ± SD)	7.2 ± 0.8	6.9 ± 1.0	0.381

### Differences between groups: T2 values and texture features

After outlier removal, statistically significant group differences were found between HC and those participants with a primary headache disorder (migraine, TTH-, and TTH+) for muscle T2 (*p* < 0.001, higher values in participants with primary headache disorders), sumaverage (*p* = 0.017, lower values in participants with primary headache disorders), kurtosisglobal (*p* = 0.024, higher values in participants with primary headache disorders), and energy (*p* = 0.043, higher values in participants with primary headache disorders). Furthermore, a statistical trend was found for skewnessglobal (*p* = 0.057, higher values in participants with primary headache disorders). The respective mean ± SD values and 95% CIs of the muscle T2 values and first- and second-order texture features can be found in Table 3. Supplementary Tables 3 & 4 show the respective results for group comparisons within the participants with primary headache disorders for first- and second-order texture features (e.g., migraine vs. TTH-, migraine vs. TTH+, TTH- vs. TTH+).

Based on ROC analyses, muscle T2 showed an AUC = 0.75 (*p* = 0.001, 95% CI: 0.613–0.877), sumaverage showed an AUC = 0.68 (*p* = 0.018, 95% CI: 0.541–0.812), kurtosisglobal showed an AUC = 0.68 (*p* = 0.024, 95% CI: 0.548–0.815), and energy showed an AUC = 0.66 (*p* = 0.043, 95% CI: 0.527–0.799).

**Table 3:** Muscle T2 and texture features from texture analysis (TA) on T2 maps from magnetic resonance imaging (MRI) of the upper trapezius muscles, expressed as mean ± standard deviation (SD) and 95% confidence intervals (CIs) for the participants with migraine, tension-type headache only (TTH-), tension-type headache plus migraine (TTH+), as well as healthy controls (HC). Outlier removal was achieved separately per each of the twelve parameters (number of removed outliers per parameter: skewnessglobal, *n* = 6; kurtosisglobal, *n* =  10; energy, *n* = 10; contrast, *n* = 1; entropy, *n* =  5; homogeneity, *n* =  7; correlation, *n* = 1; dissimilarity, *n* =  2). * statistically significant after adjustments for multiple comparisons using the Benjamini-Hochberg procedure with an accepted false discovery rate (FDR) of 15%.

	HC	Migraine / TTH- / TTH+	*p*-value
Muscle T2 (ms)	30.14 ± 1.05 [29.68;30.61]	31.10 ± 0.92 [30.83;31.36]	< 0.001*
Varianceglobal	52.93 ± 11.93 [47.64;58.22]	49.86 ± 10.87 [46.74;52.99]	0.338
Skewnessglobal	0.38 ± 0.18 [0.30;0.47]	0.49 ± 0.21 [0.43;0.56]	0.057
Kurtosisglobal	1.15 ± 0.32 [0.99;1.30]	1.63 ± 0.81 [1.37;1.88]	0.024*
Energy	0.003514 ± 0.0006 [0.003;0.004]	0.004176 ± 0.0012 [0.004;0.005]	0.043*
Contrast	40.27 ± 8.01 [36.71;43.82]	36.61 ± 10.42 [33.59;39.64]	0.145
Entropy	8.74 ± 0.33 [8.59;8.89]	8.57 ± 0.46 [8.43;8.71]	0.157
Homogeneity	0.21 ± 0.02 [0.20;0.22]	0.22 ± 0.03 [0.21;0.23]	0.107
Correlation	0.31 ± 0.05 [0.29;0.33]	0.30 ± 0.04 [0.29;0.31]	0.819
Sumaverage	0.007163 ± 0.0011 [0.007;0.008]	0.006366 ± 0.0012 [0.006;0.007]	0.017*
Variance	0.007125 ± 0.0014 [0.007;0.008]	0.006381 ± 0.0021 [0.006;0.007]	0.164
Dissimilarity	4.68 ± 0.58 [4.43;4.94]	4.43 ± 0.71 [4.22;4.64]	0.160

### Principal component analysis

**Fig. 2:**
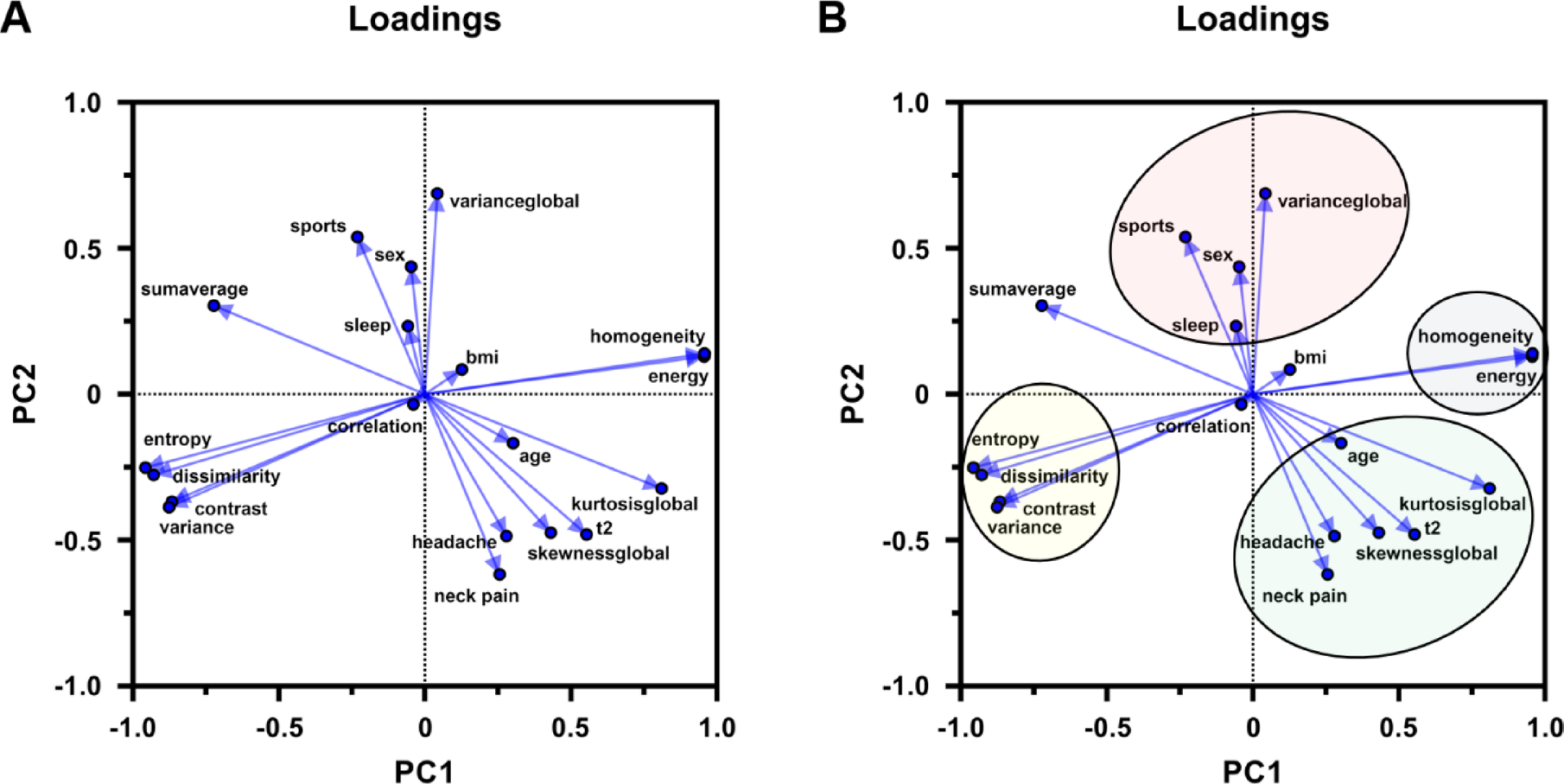
Loadings plot for principal component analysis (PCA). The numerical values from the loadings matrix are shown for principal component (PC) 1 and PC2 (**A**), together with related suggested clusters based on visual assessment (**B**). Four clusters were visually identified: age, days with headache in the last three months, neck pain, muscle T2, skewnessglobal, and kurtosisglobal (right lower quadrant); sex, average hours of sports per week, average hours of sleep per week, and varianceglobal coming close (left upper / right upper quadrant); dissimilarity, contrast, entropy, and variance (left lower quadrant); and energy and homogeneity (right upper quadrant) (**B**).

**Fig. 3:**
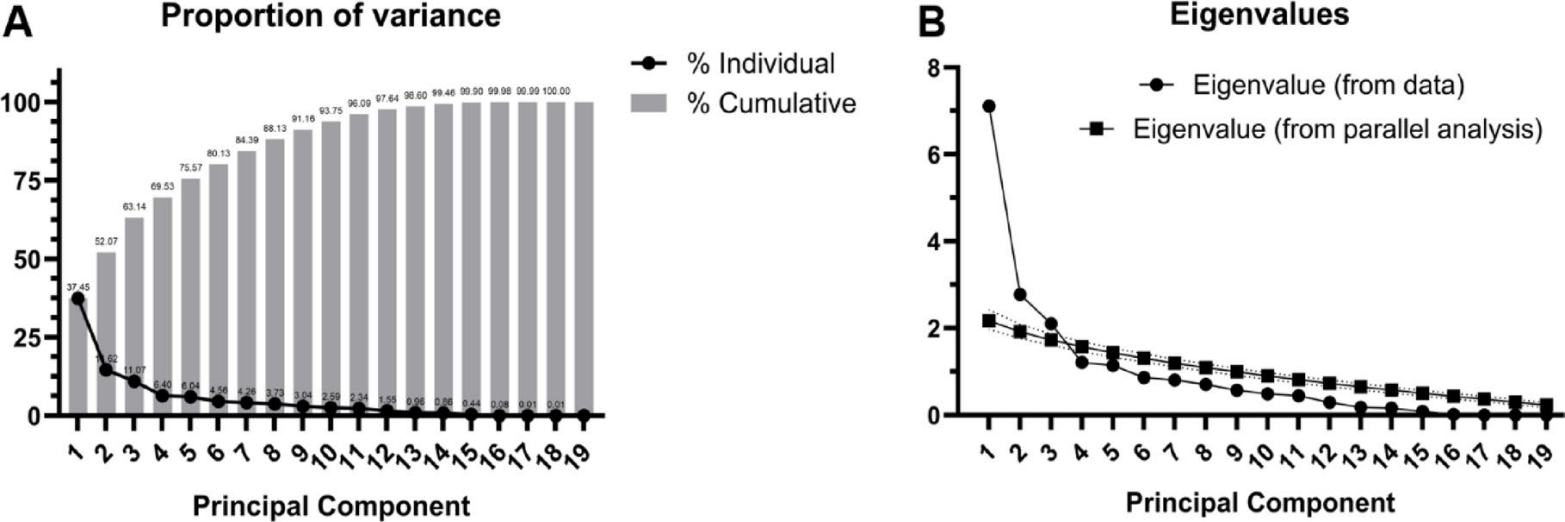
Variance and eigenvalues for principal component analysis (PCA). This figure shows the proportion of variance (**A**) and eigenvalues (**B**) for the principal components (PCs), including both the individual and cumulative proportions of variance (**A**) and eigenvalues from the data and from the parallel analysis (**B**).

According to PCA, 19 PCs were outputted in the whole cohort, with a proportion of variance / cumulative proportion of variance of 37.45% / 37.45%, 14.62% / 52.07%, and 11.07% / 63.14% (Figs. 2 and 3). Visually, regarding the loadings for PC1 and PC2, a cluster including age, days with headache in the last three months, neck pain, muscle T2, kurtosisglobal, and skewnessglobal could be observed (Fig. 2). Loadings in a cluster close to the opposite direction were average hours of sports per week, average hours of sleep per week, sex, and varianceglobal, yet the latter being closely located in the neighboring quadrant (Fig. 2). Further clusters were observed for dissimilarity, contrast, entropy, and variance, and in opposite direction for energy and homogeneity, respectively (Fig. 2). The eigenvalues, loadings, and eigenvectors are shown in Supplementary Tables 5 & 6.

### Regression analyses

In the whole cohort after removal of outliers, muscle T2 was significantly associated with days with headache in the last three months (coefficient = 3.72, *p* = 0.015). Likewise, also sumaverage (coefficient = -4689.87, *p* = 0.022) was significantly associated with days with headache in the last three months. Neither kurtosisglobal nor energy were significantly associated with headache over the last three months (*p* > 0.05).

Regarding neck pain, sumaverage was significantly associated with the presence of neck pain (coefficient = -512.44, *p* = 0.024). Furthermore, a statistical trend was found for the association between muscle T2 and neck pain (coefficient = 0.45, *p* = 0.095). Kurtosisglobal and energy were not significantly associated with neck pain (*p* > 0.05).

Furthermore, the number of mTrPs was not significantly associated with muscle T2, sumaverage, kurtosisglobal, or energy (*p* > 0.05).

## Discussion

In this study, MRI-based T2 mapping covering the upper trapezius muscles with TA was analyzed in HC and participants with migraine, TTH-, or TTH+. The main findings of this study are as follows: (1) significant group differences between HC and participants with a primary headache disorder (migraine, TTH-, and TTH+) were found for muscle T2, sumaverage, kurtosisglobal, and energy; (2) visual inspection of PCA indicated four clusters depending on the loadings regarding PC1 and PC2 (age, days with headache in the last three months, neck pain, muscle T2, skewnessglobal, and kurtosisglobal; sex, average hours of sports per week, average hours of sleep per week, and varianceglobal coming close; dissimilarity, contrast, entropy, and variance; and energy and homogeneity), (3) muscle T2 as well as sumaverage were significantly associated with the number of days with headache over the last three months, and (4) sumaverage was significantly associated with the presence of neck pain.

In the present study, MRI-based T2 mapping was performed using a high-resolution T2-prepared 3D TSE sequence, which could deliver swift and accurate T2 quantification with adequate robustness to B1 and B0 errors^21–23,37^. This may be especially relevant in challenging regions such as the neck and shoulder area, which is typically subject to relatively large B0 variations^21–23,37^. In comparison to other MRI-based T2 mapping sequences that are more frequently used, such as a multi-echo spin-echo sequence, this approach could better compensate for issues of T2 quantification in association with B1 or B0 errors^22,49,50^. The obtained T2 mapping data of this study have then been further analyzed with TA using common first-order and second-order texture features. Commonly, regarding cross-sectional in-vivo imaging, TA has been applied to standard T1- or T2-weighted sequences from MRI or computed tomography data with various purposes^43,51–54^. Regarding more advanced sequences, TA has previously been used on more advanced and quantitative MRI data, such as chemical shift encoding-based water-fat MRI with derived proton density fat fraction maps or T1 and T2 maps for myocardial tissue^39–42,55,56^. Yet, our herein presented approach of using TA on T2 maps covering the upper trapezius muscles is novel, and it reflects an additive approach using quantification from T2 mapping in combination with TA, which may have higher potential for detecting and characterizing changes in the spatial heterogeneity of upper trapezius muscles within a multi-parametric framework. Indeed, beyond significant differences between HC and participants with primary headache disorders for muscle T2, significant group differences were found for sumaverage, kurtosisglobal, and energy.

Interpreting distinct texture features and setting those into clinical context can be complex in general^57,58^. Kurtosisglobal is considered a measure of relative flatness of the histogram, with high kurtosis indicating that voxel values are concentrated around the mean (i.e., leading to a sharp peak), whereas low kurtosis may indicate flatter distributions. Significantly higher kurtosisglobal was found in participants with primary headache disorders, hence a more peak-like distribution was observed. Kurtosisglobal may be increased by intensity variations in image datasets, hence we speculate that marked T2 variations may be more present in upper trapezius muscles of patients with primary headaches. In this regard, elevated T2 may be primarily due to subtle edema, hence could be the result of processes related to neurogenic inflammation^7,24–26^. Moreover, for sumaverage derived from the GLCM, significantly lower values were seen in participants with primary headache disorders as compared to HC, with sumaverage representing the spread of the mean voxel co-occurrence distribution (i.e., average of the sum of gray-level pairs). This texture feature could quantify the texture, with high sumaverage indicating that there might be many voxel pairs with large gray-level sums, whereas low sumaverage may reflect that pairs have smaller sums. This may also correspond well to marked T2 variations in participants with primary headache disorders, given that those may have more subtle brighter appearances and, hence, lower gray-level sums. As a second-order texture feature, energy could be interpreted in the context of image uniformity or homogeneity, indicating how voxels are distributed across the GLCM. While absolute differences in values for energy were low according to group comparisons, those might still suggest differences in homogeneity of voxels between the two groups.

Given the high complexity in interpretability of texture features as well as T2 mapping as advanced methods, it seems particularly important to not see image findings in isolation, but explore potential relationships with cohort characteristics and clinical findings. Hence, MRI-derived parameters could be set into a broader context beyond imaging alone. In the present study, this has been attempted via PCA and different regression analyses. In PCA, based on visual interpretations, a cluster including age, days with headache in the last three months, neck pain, muscle T2, kurtosisglobal, and skewnessglobal was observed, and a cluster close to the opposite direction included average hours of sports per week, average hours of sleep per week, sex, and varianceglobal, yet the latter being closely located in the neighboring quadrant. Hence, directions of relationships seem to be positive for days with headache and neck pain in relation to MRI-derived parameters of muscle T2, kurtosisglobal, and skewnessglobal, with hours of sports per week as well as average hours of sleep per week being directed against those loadings. This might make sense with the observation of less sports activities and less sleep having eventually negative impact on headache, and close ties between bad sleep quality or sleep deprivation and headaches have been established before^59–62^. Likewise, regular sports activity may exert a prophylactic effect on headache occurrence and vice versa^63–66^. Our PCA could link imaging findings to those aspects, given that we can see that for loadings, muscle T2, kurtosisglobal, and skewnessglobal directed in the opposite direction as compared to average hours of sleep or sports, thus those imaging-derived measures may be inversely tied (i.e., the lesser hours of sleep or sports, the higher muscle T2, kurtosisglobal, and skewnessglobal). Moreover, our regression analyses showed that muscle T2 as well as sumaverage were significantly associated with days with headache over the last three months. Likewise, sumaverage was significantly associated with the presence of neck pain. Taken together, those results may emphasize the potential role of those image-derived parameters in the direct clinical context among primary headache disorders. The two other clusters observed included dissimilarity, contrast, entropy, and variance, and in the opposite direction energy and homogeneity, respectively, without inclusion of any demographic or headache characteristics in those clusters. Thus, one could argue that those texture features may not be ultimately meaningful in the clinical context among primary headache disorders.

As a major limitation, all participants with primary headache disorders were only investigated in their inter-ictal phases, with the distinct interval to the previous headache event not known in our analyses. Second, we also did not correlate our findings to the hormonal status of female participants, yet headaches and eventually imaging findings may be subject to changes across the menstrual cycle. Given that most of our study participants were women in their premenopausal stage of life, information on the hormonal contributions might be needed to refine our results. In this regard, also a matched study design with two or more groups closely matched in terms of characteristics such as age, sex, and further aspects such as menstrual cycle phase details would be warranted. Third, we did not evaluate any potential associations between muscle T2 values or texture features and the intensity of headache and neck pain, although such associations could further add to the relevance of muscle T2 and TA in the context of myofascial involvement and peripheral sensitization in primary headaches. In this regard, neck pain should also be investigated longitudinally over the migraine cycle and with standardized and more detailed assessment tools, such as for example a calendar for neck pain or the Neck Disability Index^67,68^. Fourth, besides assessment of mTrPs, also other findings related to neck involvement should be considered in the context of an approach to better characterize the complex clinical picture of myofascial involvement in primary headache disorders, including assessments of limited active/passive range of motion, pain during active contraction or active/passive stretching, hypersensitivity, or muscular hyper- or hypotonia with muscular imbalance, which have been previously described among primary headache disorders^7,8,20,69–72^. Fifth, in this study we primarily aimed to compare participants with primary headache disorders against HC, thus considering clinical characteristics and MRI-derived muscle T2 and texture features as a continuum across headaches. While in the context of the TCC, TTH and migraine may lie within a semiological continuum as several cranial nerves and the greater occipital nerves could represent potential sources or input for head pain^16–19^, primarily distinct diagnostic categories for single headache entities are considered within the ICHD^32^. Yet, although highly relevant and even at least common as nausea in patients with an acute episode of migraine and also in inter-ictal intervals^6,7,73,74^, the role of peripheral sensitization at the neck area with neck pain is not considered by default by the current diagnostic criteria of the ICHD^32^. Hence, future studies with larger cohorts separately for migraine and TTH with or without concomitant migraine should explore potential subgroup-specific alterations in MRI-derived texture features of the upper trapezius muscles. Such studies may also consider investigating not only the trapezius muscles, but also other muscles within and outside of the context of the TCC. By the data of the present study, it cannot be determined whether alterations in muscle T2 values and texture features in the context of primary headache disorders are exclusively detectable in the trapezius muscles, or may also be observed in other muscles especially at the neck and shoulder region.

## Conclusions

Imaging by T2 mapping of the upper trapezius muscles and TA were investigated in participants with primary headache disorders and HC. We found significant group differences between HC and participants with primary headache disorders (migraine, TTH-, and TTH+) for muscle T2, kurtosisglobal, sumaverage, and energy. Furthermore, PCA indicated a cluster including age, days with headache over the last three months, neck pain, muscle T2, kurtosisglobal, and skewnessglobal, with a cluster close to the opposite direction including average hours of sports per week, average hours of sleep per week, sex, and eventually also varianceglobal. Muscle T2 and sumaverage were significantly associated with days with headache over the last three months, while sumaverage was significantly associated with the presence of neck pain. Hence, T2 mapping with TA may provide objective quantitative parameters of the myofascial involvement and peripheral sensitization among primary headache disorders within the concept of the TCC.

## Electronic supplementary material

Below is the link to the electronic supplementary material.

Supplementary file 1

## Data Availability

The data that support the findings of this study are available from the corresponding author, but restrictions apply to the availability of these data.

## Abbreviations

3D: Three-dimensional
AUC: Area under the curve
BMI: Body mass index
CI: Confidence interval
FDR: False discovery rate
GLCM: Gray-level co-occurrence matrix
HC: Healthy control
ICHD: International Classification of Headache Disorders
MRI: Magnetic resonance imaging
mTrP: Myofascial trigger point
PC: Principal component
PCA: Principal component analysis
RMSCV: Root-mean-square coefficient of variation
ROC: Receiver operating characteristics
ROI: Region of interest
ROUT: Robust regression followed by outlier identification
SD: Standard deviation
TA: Texture analysis
TCC: Trigemino-cervical complex
TSE: Turbo spin-echo
TTH: Tension-type headache
YLD: Years lived with disability
