# Supplementary material for: Texture analysis on muscle T2 maps can reveal changes in upper trapezius muscles related to primary headache disorders
Source: Sci Rep. 2026 Jul 27;16:23353. doi: 10.1038/s41598-026-63292-7 (PMC13408834; doi:10.1038/s41598-026-63292-7)
Supplement: Supplementary file 1 [file 41598_2026_63292_MOESM1_ESM.docx]

**Supplementary Material:**

| **Repetition time (TR)** | 1,500 ms |
| --- | --- |
| **Echo time (TE)** | 16 ms |
| **Field of view (FOV)** | 480×200×84 mm^3^ |
| **Acquisition voxel** | 1.75×1.75×2.0 mm^3^ |
| **Reconstruction voxel** | 1.5×1.5×2.0 mm^3^ |
| **Echo train length** | 55 |
| **Echo spacing** | 2.3 ms |
| **Fat suppression** | Spectral inversion recovery |
| **Compressed sensitivity encoding** | Reduction factor R=5.5, SENSE |
| **T2 preparation** | 15 – 30 – 45 ms |
| **Scan time** | 7 min 53 s |

Supplementary Table 1: Sequence parameters for T2 mapping: T2‐prepared three-dimensional (3D) turbo spin-echo (TSE) sequence [21, 22].

|  | **Migraine**  (n = 21) | **TTH-**  (n = 16) | **TTH+**  (n = 12) |
| --- | --- | --- | --- |
| **Age** (mean ± SD, in years) | 24.6 ± 3.1 | 24.8 ± 3.4 | 23.6 ± 3.4 |
| **Sex** (females) | 18 (86%) | 12 (75%) | 12 (100%) |
| **BMI** (mean ± SD, in kg/m^2^) | 21.6 ± 1.9 | 22.1 ± 2.4 | 21.7 ± 1.7 |
| **Neck pain** (present) | 10 (48%) | 10 (63%) | 10 (83%) |
| **Days with headache**  (in the last three months, mean ± SD) | 14.6 ± 8.3 | 26.6 ± 24.8 | 22.0 ± 12.1 |
| **Number of mTrPs**  (all, mean ± SD) | 4.3 ± 3.4 | 3.8 ± 2.8 | 3.8 ± 2.0 |
| **Hours of sports**  (per week, mean ± SD) | 2.8 ± 1.2 | 2.8 ± 1.7 | 2.6 ± 1.6 |
| **Hours of sleep**  (per week, mean ± SD) | 6.8 ± 0.6 | 7.3 ± 1.2 | 6.7 ± 1.2 |

*Supplementary Table 2: Cohort characteristics for the participants with migraine (MIG), tension-type headache only (TTH-), and tension-type headache plus migraine (TTH+). Data are shown as mean ± standard deviation (SD) or absolute / relative frequencies.*

|  | **Migraine** | **TTH-** | **TTH+** | **p-value**  **(Migraine vs. TTH-)** | **p-value**  **(Migraine vs. TTH+)** | **p-value**  **(TTH- vs. TTH+)** |
| --- | --- | --- | --- | --- | --- | --- |
| **Varianceglobal** | 48.69 ± 11.6 | 53.46 ± 9.92 | 47.12 ± 10.36 | 0.138 | 0.782 | 0.1212 |
| **Skewnessglobal** | 0.47 ± 0.24 | 0.51 ± 0.17 | 0.51 ± 0.21 | 0.359 | 0.393 | 0.585 |
| **Kurtosisglobal** | 1.60 ± 0.85 | 1.61 ± 0.84 | 1.70 ± 0.74 | 0.900 | 0.629 | 0.643 |
| **Energy** | 0.004183 ± 0.001222 | 0.004137 ± 0.001073 | 0.004216 ± 0.001469 | 0.850 | 0.839 | 0.893 |
| **Contrast** | 36.04 ± 10.06 | 36.29 ± 8.27 | 38.03 ± 13.77 | 0.635 | 0.542 | 0.792 |
| **Entropy** | 8.56 ± 0.46 | 8.49 ± 0.52 | 8.71 ± 0.38 | 0.961 | 0.502 | 0.397 |
| **Homogeneity** | 0.22 ± 0.03 | 0.22 ± 0.03 | 0.22 ± 0.03 | 0.900 | 0.403 | 0.585 |
| **Correlation** | 0.30 ± 0.05 | 0.30 ± 0.04 | 0.30 ± 0.04 | 0.612 | 0.868 | 0.548 |
| **Sumaverage** | 0.006717 ± 0.001031 | 0.006173 ± 0.001314 | 0.006010 ± 0.001226 | 0.195 | 0.124 | 0.741 |
| **Variance** | 0.006378 ± 0.001897 | 0.006139 ± 0.002306 | 0.006711 ± 0.002362 | 0.9699 | 0.599 | 0.698 |
| **Dissimilarity** | 4.37 ± 0.73 | 4.41 ± 0.62 | 4.58 ± 0.83 | 0.635 | 0.481 | 0.610 |

*Supplementary Table 3: Texture features from texture analysis (TA)* *on T2 maps from magnetic resonance imaging (MRI) of the upper trapezius muscles, expressed as mean ± standard deviation (SD) for the participants with migraine, tension-type headache only (TTH-), and tension-type headache plus migraine (TTH+). Outlier removal was achieved separately per each of the twelve parameters. None of the comparisons showed statistically significant results (p>0.05).*

|  | **HC** | **TTH-** | **Migraine / TTH+** | **p-value**  **(HC vs. TTH-)** | **p-value**  **(HC vs. Migraine / TTH+)** | **p-value**  **(TTH- vs. Migraine / TTH+)** |
| --- | --- | --- | --- | --- | --- | --- |
| **Varianceglobal** | 52.93 ± 11.93 | 53.46 ± 9.92 | 48.12 ± 11.03 | 0.737 | 0.125 | 0.079 |
| **Skewnessglobal** | 0.38 ± 1.18 | 0.51 ± 0.17 | 0.49 ± 0.23 | 0.051 | 0.136 | 0.672 |
| **Kurtosisglobal** | 1.15 ± 0.32 | 1.61 ± 0.84 | 1.63 ± 0.80 | 0.114 | 0.028 | 0.927 |
| **Energy** | 0.003514 ± 0.0006 | 0.004137 ± 0.001073 | 0.004194 ± 0.001286 | 0.082 | 0.076 | 0.941 |
| **Contrast** | 40.27 ± 8.01 | 36.29 ± 8.27 | 36.76 ± 11.37 | 0.334 | 0.153 | 0.843 |
| **Entropy** | 8.74 ± 0.33 | 8.49 ± 0.52 | 8.61 ± 0.43 | 0.180 | 0.249 | 0.748 |
| **Homogeneity** | 0.21 ± 0.02 | 0.22 ± 0.03 | 0.22 ± 0.03 | 0.198 | 0.147 | 0.888 |
| **Correlation** | 0.31 ± 0.05 | 0.30 ± 0.04 | 0.30 ± 0.04 | 0.658 | 0.956 | 0.523 |
| **Sumaverage** | 0.007163 ± 0.0011 | 0.006173 ± 0.001314 | 0.006460 ± 0.001140 | 0.026 | 0.047 | 0.456 |
| **Variance** | 0.007125 ± 0.0014 | 0.006139 ± 0.002306 | 0.006499 ± 0.002048 | 0.204 | 0.240 | 0.879 |
| **Dissimilarity** | 4.68 ± 0.58 | 4.41 ± 0.62 | 4.44 ± 0.76 | 0.319 | 0.180 | 0.919 |

*Supplementary Table 4: Texture features from texture analysis (TA)* *on T2 maps from magnetic resonance imaging (MRI) of the upper trapezius muscles, expressed as mean ± standard deviation (SD) for the participants with migraine, tension-type headache only (TTH-), and a collapsed group of tension-type headache plus migraine (TTH+) and migraine. Outlier removal was achieved separately per each of the twelve parameters. None of the comparisons showed statistically significant results after adjustments for multiple comparisons using the Benjamini-Hochberg procedure with an accepted false discovery rate (FDR) of 15% (performed three times, i.e. within each group comparison separately).*

| **Principal Component** | **Eigenvalue (from data)** | **Eigenvalue (from parallel analysis)** | | |
| --- | --- | --- | --- | --- |
|  | **Mean** | **Mean** | **Upper Limit** | **Lower Limit** |
| **PC1** | 7.1153 | 2.1750 | 2.4309 | 1.9796 |
| **PC2** | 2.7781 | 1.9201 | 2.0891 | 1.7711 |
| **PC3** | 2.1024 | 1.7329 | 1.8668 | 1.6084 |
| **PC4** | 1.2153 | 1.5767 | 1.6949 | 1.4629 |
| **PC5** | 1.1477 | 1.4381 | 1.5465 | 1.3367 |
| **PC6** | 0.8663 | 1.3140 | 1.4145 | 1.2220 |
| **PC7** | 0.8091 | 1.1997 | 1.2887 | 1.1130 |
| **PC8** | 0.7095 | 1.0956 | 1.1817 | 1.0128 |
| **PC9** | 0.5772 | 0.9970 | 1.0775 | 0.9158 |
| **PC10** | 0.4912 | 0.9017 | 0.9796 | 0.8241 |
| **PC11** | 0.4452 | 0.8148 | 0.8903 | 0.7399 |
| **PC12** | 0.2940 | 0.7316 | 0.8073 | 0.6570 |
| **PC13** | 0.1828 | 0.6530 | 0.7241 | 0.5817 |
| **PC14** | 0.1638 | 0.5786 | 0.6467 | 0.5106 |
| **PC15** | 0.0831 | 0.5066 | 0.5743 | 0.4401 |
| **PC16** | 0.0148 | 0.4370 | 0.5006 | 0.3760 |
| **PC17** | 0.0023 | 0.3694 | 0.4340 | 0.3092 |
| **PC18** | 0.0016 | 0.3020 | 0.3665 | 0.2426 |
| **PC19** | 0.0002 | 0.2304 | 0.2920 | 0.1697 |

*Supplementary Table 5: Eigenvalues (mean and upper and lower limits) for principal component analysis (PCA) with 19 principal components (PCs; PC1 – PC19). The eigenvalues are shown as a result from the data as well as the parallel analysis.*

| **Variables** | **Loadings** | | **Eigenvectors** | |
| --- | --- | --- | --- | --- |
|  | **PC1** | **PC2** | **PC1** | **PC2** |
| **Age** | 0.3019 | -0.1676 | 0.1132 | -0.1006 |
| **Sex** | -0.0476 | 0.4368 | -0.0178 | 0.2621 |
| **BMI** | 0.1265 | 0.0848 | 0.0474 | 0.0509 |
| **Neck_pain** | 0.2557 | -0.6177 | 0.0958 | -0.3706 |
| **Headache_days (3months)** | 0.2790 | -0.4862 | 0.1046 | -0.2917 |
| **Sports_hours (week)** | -0.2315 | 0.5388 | -0.0868 | 0.3232 |
| **Sleep_hours (week)** | -0.0583 | 0.2334 | -0.0219 | 0.1400 |
| **T2** | 0.5535 | -0.4808 | 0.2075 | -0.2885 |
| **Varianceglobal** | 0.0423 | 0.6880 | 0.0159 | 0.4128 |
| **Skewnessglobal** | 0.4309 | -0.4745 | 0.1615 | -0.2847 |
| **Kurtosisglobal** | 0.8103 | -0.3231 | 0.3038 | -0.1938 |
| **Energy** | 0.9563 | 0.1291 | 0.3585 | 0.0775 |
| **Contrast** | -0.8668 | -0.3687 | -0.3250 | -0.2212 |
| **Entropy** | -0.9577 | -0.2518 | -0.3590 | -0.1511 |
| **Homogeneity** | 0.9573 | 0.1393 | 0.3589 | 0.0836 |
| **Correlation** | -0.0398 | -0.0348 | -0.0149 | -0.0209 |
| **SumAverage** | -0.7230 | 0.3036 | -0.2711 | 0.1822 |
| **Variance** | -0.8765 | -0.3871 | -0.3286 | -0.2322 |
| **Dissimilarity** | -0.9291 | -0.2767 | -0.3483 | -0.1660 |

*Supplementary Table 6: Loadings and eigenvectors for principal component analysis (PCA) with the first two principal components (PCs; PC1 & PC2).*
